# Tuning of ion-release capability from bio-ceramic-polymer composites for enhancing cellular activity

**DOI:** 10.1098/rsos.190612

**Published:** 2019-09-11

**Authors:** Naoki Osada, Arisa Terada, Hirotaka Maeda, Akiko Obata, Yasutoshi Nishikawa, Toshihiro Kasuga

**Affiliations:** 1 Nagoya Institute of Technology, Gokiso cho, Showa ku, Nagoya 466-8555, Japan; 2 ORTHOREBIRTH Co. Ltd, 15-3-303 Chigasaki-Chuo, Tsuzuki-ku, Yokohama 224-0032, Japan

**Keywords:** biomaterial, composite, glass, ion-release, poly(lactic-*co*-glycolic acid), calcium carbonate

## Abstract

In our previous study, we investigated the synergetic effects of inorganic ions, such as silicate, Mg^2+^ and Ca^2+^ ions on the osteoblast-like cell behaviour. Mg^2+^ ions play an important role in cell adhesion. In the present study, we designed a new composite that releases a high concentration of Mg^2+^ ions during the early stage of the bone-forming process, and silicate and Ca^2+^ ions continuously throughout this process. Here, 40SiO_2_–40MgO–20Na_2_O glass (G) with high solubility and vaterite-based calcium carbonate (V) were selected as the source of silicate and Mg^2+^ and Ca^2+^ ions, respectively. These particles were mixed with poly(lactic-*co*-glycolic acid) (PLGA) using a kneading method at 110°C to prepare the composite (G-V/PLGA, G/V/PLGA = 4/56/40 (in weight ratio)). Most of the Mg^2+^ ions were released within 3 days of immersion at an important stage for cell adhesion, and silicate and Ca^2+^ ions were released continuously at rates of 70–80 and 180 ppm d^−1^, respectively, throughout the experiment (until day 7). Mouse-derived osteoblast-like MC3T3-E1 proliferated more vigorously on G-V/PLGA in comparison with V-containing PLGA without G particles; it is possible to control the ion-release behaviour by incorporating a small amount of glass particles.

## 1. Introduction

Tissue engineering combining three elements: namely, cells, scaffolds and growth factors, has been attracting attention in recent years as a method for repairing tissues damaged from injury or disease [1]. Examples of the growth factor include proteins typified by bone morphogenetic protein, amino acids and inorganic ions. These proteins and amino acids have many problems such as the need to be supported and released without a loss in their activity [2]. Inorganic ions are attracting attention as an inducing initiator because there is no denaturation during material synthesis. Silicate ions are known as essential elements for metabolic processes involved in bone formation [3]. Silicate ions have been reported to promote the proliferation, differentiation and calcification of osteoblast-like cells [4–10]. Ca^2+^ ions are significant components in bone and are an indispensable element for bone formation. It has been reported that Ca^2+^ ions also promote the proliferation, differentiation and calcification of osteoblast-like cells [11,12].

Poly(l-lactic acid) (PLLA) has been reported as a biodegradable polymer for bone repair [13–15]. Our group focused on silicate and calcium ions, and prepared siloxane containing vaterite (SiV) for their release [16]. PLLA composites containing SiV have been reported to promote bone formation [17], and have been successfully shaped into a cotton-wool-like form through electrospinning [18], namely, the adhesion of osteoblast-like cells starts around their cross-linking sites, and the cells penetrate between the fibres [19]. Our group has also developed a new type of cotton-wool-like, fibrous material containing SiV and β-tricalcium phosphate (β-TCP) particles. This material has shown excellent bone formation in *in vivo* tests using New Zealand white rabbits [20]. Because the PLLA is present as a matrix phase in the fibre, SiV on the surface dissolves during an early stage, releasing calcium and silicate ions. β-TCP particles were expected to be dissolved extremely slowly in the body, providing positive effects within a relatively long period after implantation. Their dissolution was controlled slowly through the decomposition of the PLLA matrix. PLLA, which does not exhibit rapid degradability, can maintain the fibrous shape during bone regeneration.

The degradability of the polymer matrix phase can be controlled using poly(lactic-*co*-glycolic acid) (PLGA) by changing the ratio of lactic acid and glycolic acid [21]. PLGAs, which are basically hydrophobic, show a swelling ability when placed in water, originating from their high degradability [22,23]. Our group reported that the PLGA composites containing calcium carbonate particles showed a continuous release of calcium ions [24]. When PLGA is compounded with an inorganic material, that is, the rapid hydrolysis of PLGA is expected to induce the effective release of therapeutic ions such as Ca^2+^ and silicate ions. PLGA might be an excellent matrix polymer for preparing a composite with a higher osteogenesis promoting effect.

Mg^2+^ ions are also essential elements for bone metabolism [25]. It has been reported that, among osteoblast-like cell mineralization processes, such ions play an important role in adhesion, acting on osteoblast integrins [25]. In recent years, the influence of the combination of multiple ions on the cell behaviour has been investigated [26–28]. In our group, when osteoblast-like cells were cultured in a medium in which the particular concentrations of Ca^2+^, silicate and Mg^2+^ ions were increased, their proliferation, differentiation and mineralization were promoted [29–31]. A typical concentration for enhancing the mineralization is Ca of 140 ppm, Si of 58 ppm, Mg of 71 ppm and P of 36 ppm (Ca of 78 ppm, Si of 0 ppm, Mg of 28 ppm and P of 40 ppm in a normal alpha minimum essential medium (α-MEM)).

The authors considered that, to induce high osteoblast activities, it is necessary to continuously supply Mg^2+^ ions, silicate ions during the initial stage and a large amount of Ca^2+^ ions after implantation of the materials. Composites using PLGA will be a key to providing a therapeutic ion-release capability.

Our group prepared a composite material using PLGA and SiV, and investigated its ion-releasing behaviour [32]. Although the continuous release of calcium ions from SiV/PLGA over a long period was observed, most of the silicate ions were released within 1 day. The water-uptake ability of PLGA is considered to form an ion-releasing pathway [24]. Amorphous siloxane bonds weakly around the primary particles of vaterite, which easily dissociates to be released in an aqueous solution [32,33].

Bioglass^®^ (45S5, 46.1SiO_2_–26.9CaO–24.4Na_2_O–2.6P_2_O_5_ in mol% [34–36], developed by Hench) is an alkaline that immediately occurs within the vicinity of the surface after implantation in the body because calcium and sodium ions are exchanged with protons in water, forming a silanol group when in contact with bodily fluids. As a result, soluble silica of Si(OH)_4_ is produced, a portion of which condenses again to form a silica gel phase. Some of the soluble silica is continuously released. Silicate glasses containing MgO are promising sources for the release of silicate and magnesium ions.

In this study, to control the release of inorganic ions in enhancing bone formation, SiO_2_–MgO–Na_2_O glass and calcium carbonate (vaterite) particles were embedded in a PLGA matrix phase. The objectives were to examine the ion-releasing behaviour and cell proliferation of the prepared composite materials and to discuss their possibility as a new bone regeneration material.

## 2. Material and methods

### 2.1. Preparation of SiO_2_–MgO–Na_2_O glass

A batch mixture of nominal compositions of 40SiO_2_–40MgO–20Na_2_O in mol% was prepared using reagent-grade chemicals such as SiO_2_, MgO and Na_2_CO_3_ (Kishida Chemical, Japan). The mixture was melted at 1500°C for 30 min in a platinum crucible in air and then quenched on stainless steel using an iron-pressing method, resulting in the formation of plate-likes glass pieces. The resulting glasses are denoted as ‘G’. The glasses were crushed roughly using an alumina mortar and then pulverized using ball-milling for 24 h with 400 g of zirconia balls of 3 mm in diameter and 30 g of glass with 100 ml of methanol.

**Figure 1.**
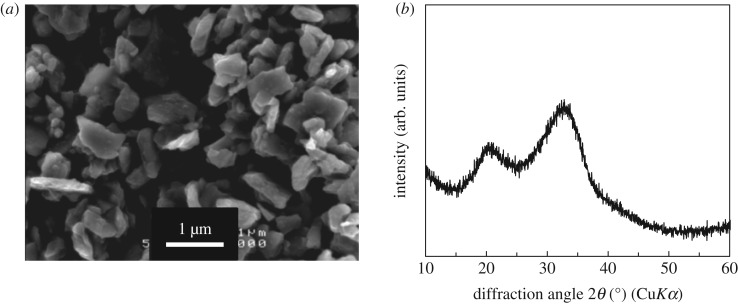
(*a*) SEM image and (*b*) XRD pattern of G particles.

Figure 1*a* shows an image of the G particles using scanning electron microscopy (SEM) (JSM-6301F, JEOL, Japan). A conductive coating treatment was carried out prior to the observation using an osmium coater (NEOC Neo Osmium Coater, Meiwafosis Co., Ltd, Japan). The glass particle sizes were approximately 1 µm or less. As shown in figure 1*b*, halo peaks were observed in an X-ray diffraction (XRD; X'pert, Philips, the Netherlands: Cu*Kα*, 45 kV, 40 mA) pattern of the particles; G was concluded to be glassy particles. In this pattern, two halo peaks were shown; this glass may contain two different networks.

The structure of the prepared glass sample was measured using laser Raman spectroscopy with an Nd: YAG laser (NRS-3300, 532 nm, JASCO Co., Japan). The structure of silicon in the glass was examined through magic-angle-spinning nuclear magnetic resonance spectroscopy (MAS-NMR) (JNM-ECA600II, JEOL, Japan) at a resonance frequency of 119.24 MHz, operating at pulses of 5.0 µs and at a recycle delay of 120 s using an 8.0 mm zirconia rotor. The sample spinning speed was 6 kHz. In addition, 4-dimethyl 4-silapentane sulfonate sodium (1.534 ppm) was used as a reference material of ^29^Si.

### 2.2. Preparation of vaterite particles

**Figure 2.**
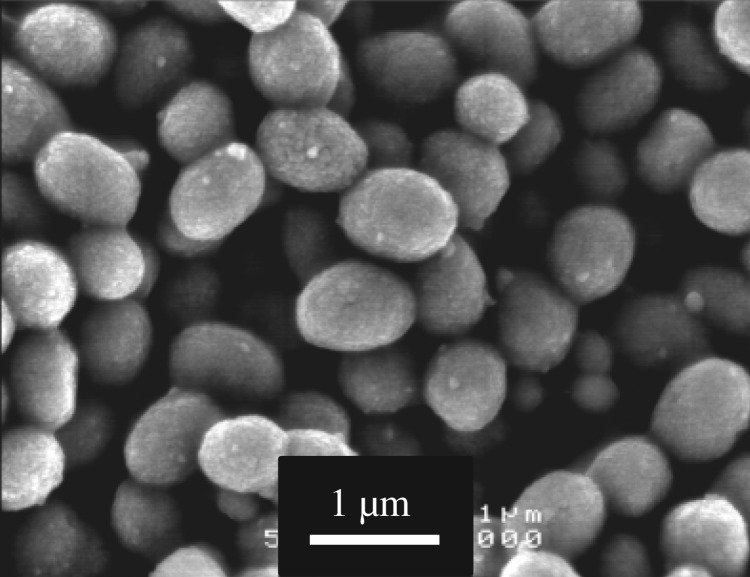
SEM image of V particles.

Calcium carbonates consisting predominantly of vaterite were prepared using a carbonation process in methanol [37]. CO_2_ gas was blown for 3 h at a flow rate of 300 ml min^−1^ into a suspension consisting of 7.0 g of Ca(OH)_2_ in 180 ml of methanol at 0°C in a Pyrex beaker. The resulting slurry was dried at 70°C in air, resulting in the formation of fine-sized powders. Figure 2 shows an SEM photograph of the powders. The surface area of the calcium carbonates obtained was determined to be 40 m^2^ g^−1^ through a nitrogen gas sorption analysis. Secondary particles of 0.5–1 mm in diameter were formed, and an agglomeration of primary particles of 20–100 nm in diameter was observed. XRD analysis (Cu*Kα*, 45 kV, 40 mA) showed that the calcium carbonate consists predominantly of vaterite with a trace amount of calcite (denoted as V hereafter).

### 2.3. Preparation of PLGA composites

To prepare a composite containing G and V, PLGA (75% lactide/25% glycolide) (PURASORB^®^, Corbion Purac, The Netherlands) with an average molecular weight (*M_w_*) of 102 kDa was used. PLGA was melted at 135°C in a kneader, and then mechanically blended with G and V particles for 10 min. The resulting composite is denoted as G-V/PLGA. For comparison, composites consisting of V and PLGA (denoted by V/PLGA) and consisting of β-TCP and PLGA (denoted by TCP/PLGA) were also prepared using the same procedure as G-V/PLGA. The composition of each sample is shown in table 1.

**Table 1.** Compositions of the prepared composites.

sample code	composition (wt%)
G-V/PLGA	G: 4, V: 56, PLGA: 40
V/PLGA	V: 60, PLGA: 40
TCP/PLGA	β-TCP: 60, PLGA: 40

Each composite was dissolved in chloroform. The composite slurry was cast in a Teflon^®^ vessel and dried at room temperature to form a film. Film-like samples were then used for the following evaluation tests.

### 2.4. Measurement of ion release from composites

The amounts of ions released from the G-V/PLGA films (14 mm in diameter and 160 ± 20 µm in thickness) were evaluated by immersing them in 1 ml of a culture medium (α-MEM) containing 10% fetal bovine serum (FBS) and incubating at 37°C in a humidified atmosphere of 95% air with 5% CO_2_ for 7 days. The medium was changed after 1 day of culturing and then changed every other day.

An XRD analysis (Cu*Kα*, 45 kV, 40 mA) of G-V/PLGA was conducted to examine the change in crystal phase before and after immersion in α-MEM.

The supernatant solution after immersion was diluted 10 times with distilled water, and the concentration of each ion in the solution was measured using inductively coupled plasma atomic emission spectrometry (ICP-AES) (ICPS-7000, Shimadzu, Japan).

To examine the charge of the ion distribution in G-V/PLGA, the composite was immersed in α-MEM for 7 days, and to prepare the sample for observation with a scanning transmission electron microscope (STEM) (JEM-2100F, JEOL, Japan) equipped with an energy dispersive X-ray spectrometer, the composite was processed using a focused ion beam processing observation apparatus (FIB) (EM-9320FIB, JEOL, Japan). An element mapping image of a section of the sample was obtained.

### 2.5. Cell culture test

PLGA, G-V/PLGA, V/PLGA and TCP/PLGA films of 14 mm in diameter and 160 ± 20 μm in thickness were prepared for the cell culture tests. They were sterilized using ethylene oxide gas. Mouse osteoblast-like cells (MC3T3-E1 cells) were seeded onto the films in 24-well plates at a density of 30 000 cells well^−1^. The α-MEM containing 10% FBS was used as the culture medium, and the cells were cultured at 37°C in 5% CO_2_ for 7 days. The medium was changed after 1 day of culturing and then changed every other day.

The number of MC3T3-E1 cells was evaluated after treatment using a Cell Counting Kit-8 (Dojindo, Japan). One reagent used in the kit is a water-soluble tetrazolium salt, which is reduced by dehydrogenases in live cells to generate a water-soluble formazan. The water-soluble formazan reaches the maximum absorption at approximately 460 nm. The numbers of live cells in the samples were counted by measuring the absorbance of the resulting medium at 450 nm using an absorption microplate reader (Sunrise Remote, TECAN Japan, Japan). The concentration of ions in the medium after culturing was measured using ICP-AES.

## 3. Results

### 3.1. Structure of G particles

**Figure 3.**
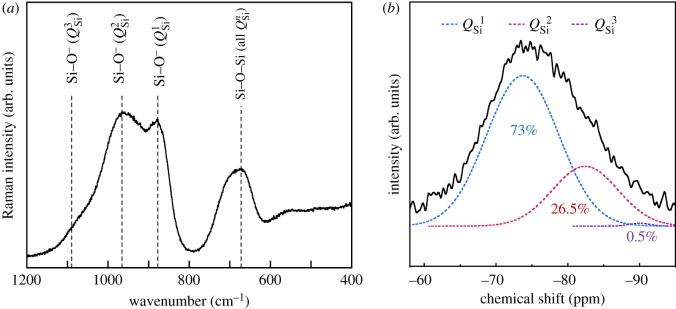
(*a*) Laser Raman and (*b*) ^29^Si MAS-NMR spectra of G particles.

Figure 3*a* shows the laser Raman spectrum of the G particles. The peaks derived from *Q*_Si_^1^, *Q*_Si_^2^ and *Q*_Si_^3^ were reported to appear at approximately 650 and 880 cm^−1^, 650 and 970 cm^−1^ and 1080 cm^−1^, respectively [38–40]. The *Q*_Si_^3^ peak was unclear.

Figure 3*b* shows the spectrum of 29Si MAS-NMR and the deconvoluted peaks using a Gaussian function. The peaks derived from *Q*_Si_^1^, *Q*_Si_^2^ and *Q*_Si_^3^, which were confirmed to appear at −73, −82 and −91 ppm, respectively, were observed [39].

The peak-integrated ratio of *Q*_Si_^1^/*Q*_Si_^2^ was estimated to be approximately 73/27. As a result, the *Q*_Si_^1^ unit was concluded to be a dominant structure without the *Q*_Si_^3^ unit.

### 3.2. Ion release from G-V/PLGA

**Figure 4.**
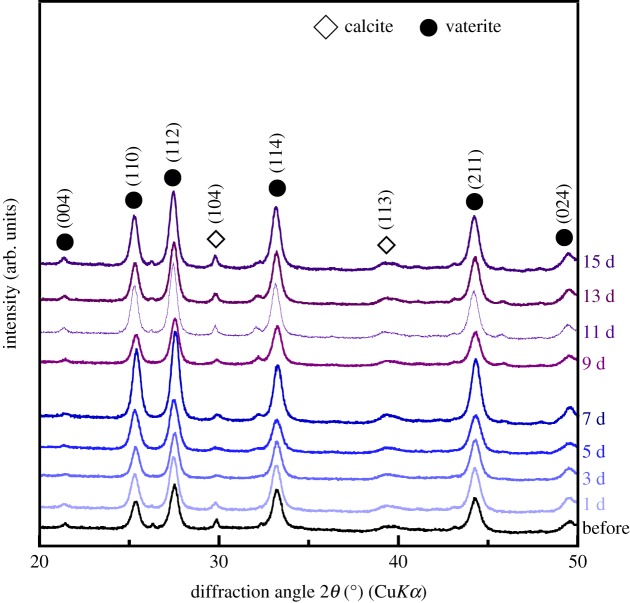
XRD patterns of G-V/PLGA before and after immersion in α-MEM.

Figure 4 shows the XRD patterns of G-V/PLGA before and after immersion in α-MEM. The peaks derived from vaterite were confirmed. A weak peak corresponding to calcite appearing in the pattern was confirmed.

**Figure 5.**
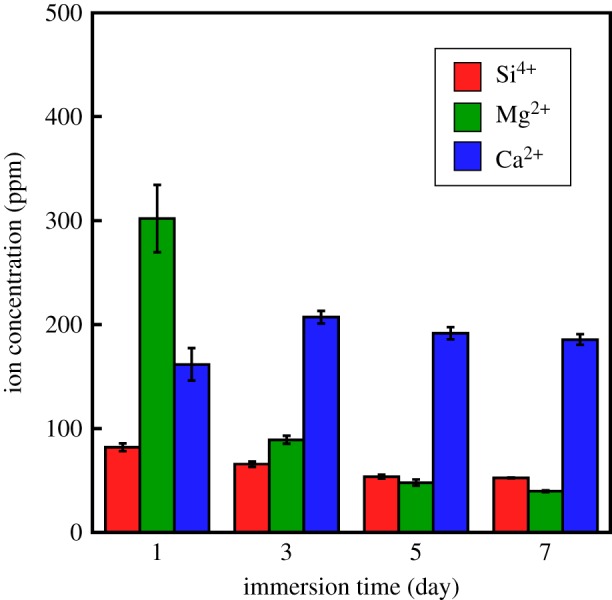
Ion-release amounts from G-V/PLGA in α-MEM. An immersion time of ‘1’ means ‘0–1’ day, whereas ‘3,’ ‘5,’ and ‘7’ indicate ‘2–3,’ ‘4–5,’ and ‘6–7’ days, respectively.

Figure 5 shows the ion release behaviour from G-V/PLGA in α-MEM. The Mg^2+^ and Ca^2+^ ions in the media were 28 and 160 ppm, respectively. Silicate ions were released continuously (approx. 70–80 ppm) from the composite during the 7-day period. Approximately 74% of the Mg^2+^ ions were released within 1 day, and approximately 90% were released within 3 days. Ca^2+^ ions were released continuously, similarly as the silicate ions.

**Figure 6.**
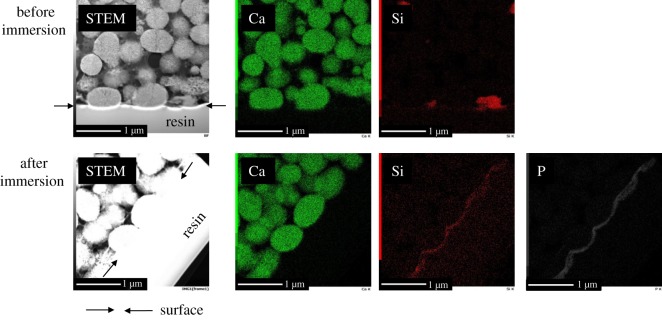
Element mapping image of cross-sectional G-V/PLGA before and after 7 days of immersion in α-MEM. The samples were prepared by FIB processing after being embedded in ‘resin’. The arrow indicates the surface of the composite.

Figure 6 shows element mapping images of the cross-section of G-V/PLGA around its surface before and after the 7 days of immersion. In the images prior to immersion, V-derived Ca and G-derived Si were observed. Note that the amount of G was only 4%. The G particles were sparsely embedded in the PLGA matrix phase. In the images taken after 7 days of immersion, V-derived Ca was clearly observed; almost no change in the distribution state was observed before or after immersion. By contrast, from the images taken after 7 days of immersion, no G particles were observed inside the material, and Si and P appeared within the vicinity of the sample surface on the V particles.

### 3.3. Proliferation of MC3T3-E1 cells

**Figure 7.**
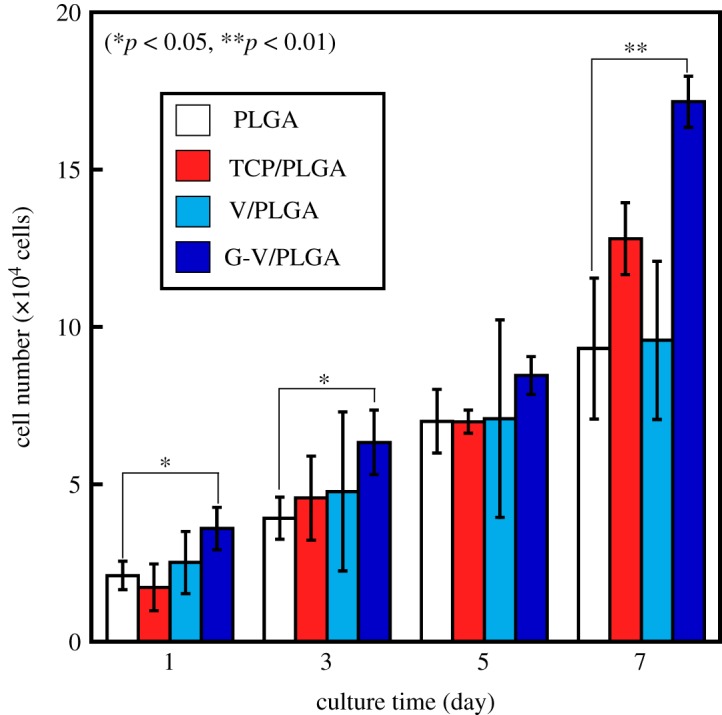
Numbers of MC3T3-E1 cells after culturing on PLGA, TCP/PLGA, V/PLGA and G-V/PLGA for 7 days.

Figure 7 shows the numbers of MC3T3-E1 cells after culturing on PLGA, TCP/PLGA, V/PLGA and G-V/PLGA for 7 days. In all samples, an increase in the number of cells was observed. G-V/PLGA showed a significantly higher number of viable cells compared with PLGA.

**Figure 8.**
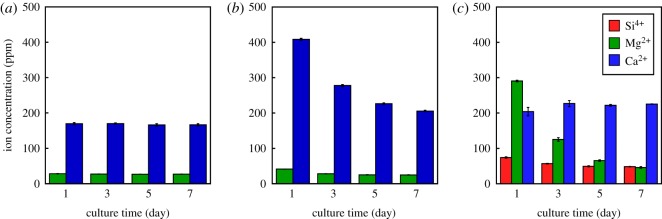
Ion-release behaviours after being cell-cultured in α-MEM from (*a*) TCP/PLGA, (*b*) V/PLGA and (*c*) G-V/PLGA. A culture time of ‘1’ indicates ‘0–1’ day, and ‘3,’ ‘5’, and ‘7’ indicate ‘2–3’, ‘4–5’, and ‘6–7’ days, respectively.

Figure 8 shows the amounts of ions released in α-MEM from TCP/PLGA, V/PLGA and G-V/PLGA after the culturing. In the case of TCP/PLGA, almost no changes in the amounts of Mg^2+^ and Ca^2+^ ions in the media after the culturing could be observed. In the case of V/PLGA, the release of a large of amount of Ca^2+^ ions was observed within 1 day, and the amounts released decreased with an increase in the culture time. In the case of G-V/PLGA, most of the Mg^2+^ ions were released within 3 days, and silicate and Ca^2+^ ions were continuously released as well, as shown in figure 5.

## 4. Discussion

G included approximately 70% of *Q*_Si_^1^ and 30% of *Q*_Si_^2^. In general, it may be difficult to prepare glasses containing almost no *Q*_Si_^2^*, Q*_Si_^3^ or *Q*_Si_^4^. Figure 1*b* may imply two types of network structures in the glass. It has been reported that, in 49.5SiO_2_–1.1P_2_O_5_–23.0MgO–26.4Na_2_O glass, approximately 85% of MgO act as network modifiers and approximately 15% as network formers, MgO_4_ units [41]. Because MgO, which is an intermediate oxide, is contained in large amounts, some of them are considered to act as a member in glass network formers facilitating the vitrification.

The Si–O–Si bond of *Q*_Si_^1^ is easily broken through hydrolysis, creating soluble silanol groups. This glass is expected to be effective as a source of release of Mg^2+^ and silicate ions.

As shown in Figure 8*c*, when the cells were cultured in G-V/PLGA, almost all Mg^2+^ ions in the G particles were released within 5 days, whereas silicate ions of 30–40 ppm d^−1^ and Ca^2+^ ions of approximately 50 ppm d^−1^ were continuously released, except for an initial burst release within day 1. The following three factors are considered reasons for these ions-release behaviours.

The first factor is the solubility of the glass particles consisting of *Q*_Si_^1^ (73%) and *Q*_Si_^2^ (27%). The short chain structure is easily broken by H_2_O through a mechanism close to the reaction of a Bioglass^®^ surface [42]. To form a silanol group on the surface, the glass releases Mg^2+^ and Na^+^ ions through an exchange with protons (or H_3_O^+^) in the medium, and a loss of soluble silica in the form of Si(OH)_4_ will then be induced. Figure 6 indicates that, after the immersion of G-V/PLGA in the medium, the G particles are dissolved, and a thin Si-rich layer around the composite surface is formed. The layer will be a silica gel phase formed through the condensation and repolymerization of the silanols. As shown in figure 6, phosphate ions appeared within the vicinity of the sample surface after immersion in α-MEM. The silica gel layer could enhance the formation of calcium phosphate phase around the sample surface. The difference in ion-releasing behaviours between G-V/PLGA and V/PLGA in figure 8 is related to the formation of the silica layer, which inhibits the initial burst release of Ca^2+^ ions from V/PLGA.

The second factor is the water-uptake capability of PLGA. PLGA has been reported to show a much higher water-uptake ability than PLLA when it contains 20% of poly(glycolic acid) and when PLLA is immersed in a phosphate buffer solution [23]. Owing to this ability of PLGA, the glass particles will be dissolved by water invading inside the material. Pathways through which the ions are released are created by this entrapped water. As a result, Mg^2+^ and Na^+^ ions are quickly released. Silicate ions are released slowly, however, owing to their low solubility, and some remain around the surface of the composite. V particles are also dissolved in the uptake water, and Ca^2+^ ions are continuously released.

The third factor is the high content of the filler, namely, 60 wt%, in the composite. In our previous study, the ion-release behaviours of the PLLA composites containing 30 and 60 wt% of SiV in a Tris buffer solution were examined [32]. The composite containing 30 wt% of SiV released approximately 60% of the silicate ions within 1 day of immersion, whereas the composite containing 60 wt% of SiV released one almost completely within 1 day. This burst release is considered to be from the percolation effect of the filler, namely SiV, owing to its contact in the PLLA matrix phase. Such an effect occurs because G-V/PLGA contains 60 wt% (approx. 50 vol%) of filler (G and V particles).

The combination of these three factors indicates that water can easily infiltrate the G-V/PLGA. The high solubility of glass implies that the infiltrated water will easily dissolve the Mg^2+^ and silicate ions, even if present in a small amount, namely, only 4 wt%. The large amount of V particles, namely, 50 wt%, indicates a continuous supply of Ca^2+^ ions. The proliferation of MC3T3-E1 on G-V/PLGA showed significantly higher values during each culture period. As shown in figure 8, the release of Ca^2+^ ions from V/PLGA, and the release of silicate, Mg^2+^ and Ca^2+^ ions from G-V/PLGA, was observed.

The combination of Mg^2+^, Ca^2+^ and silicate ions released from G-V/PLGA might enhance the proliferation of the cells. It is difficult at this stage to conclude the enhancing effect and its mechanism. However, we believe that the present study demonstrates that the inclusion of a small amount of ion-releasing glass particles might have a positive effect on the stimulation of osteoblast-like cells.

## 5. Conclusion

In this study, we aimed at the preparation of biodegradable polymer composites for tuning the release of silicate, Mg^2+^ and Ca^2+^ ions using highly soluble glass and calcium carbonate particles.

A 40SiO_2_–40MgO–20Na_2_O glass with high solubility was newly prepared as the releasing source of Mg^2+^ and silicate ions. The glass consisted predominantly of a *Q*_Si_^1^ group with a portion of a *Q*_Si_^2^ group; this structure was shown to ease the dissolution in an aqueous solution.

A total of 4 wt% of glass particles and 56% calcium carbonate (vaterite) particles containing a trace amount of calcite were embedded in a PLGA matrix phase. The resulting composite showed a unique ion-releasing behaviour in a culture medium, demonstrating a burst release of Mg^2+^ ions immediately after immersion in the medium and the continuous release of Ca^2+^ and silicate ions.

Such an ion dissolution behaviour is thought to be caused by the following three factors: (i) the high solubility of the glass particles, (ii) the high water-uptake capability of PLGA and (iii) the percolation effect of the filler (60 wt%) embedded in the polymer matrix.

The continuous release of silicate ions was considered to originate from the balance between the amounts of soluble silica and the gelating one derived from the hydrolysis of the glass particles. The continuous dissolution of Ca^2+^ ions originated from the high solubility of vaterite and a percolation effect with a large amount of filler, namely, 60 wt%, in addition to the hydrous capability of PLGA.

A tuning of the ion-release behaviour when using a small amount of glass particles demonstrated the possibility of enhancing the proliferation of osteoblast-like cells.

## Supplementary Material

The raw data of each figure

Reviewer comments

## Data accessibility

The datasets supporting this article have been uploading as part of the electronic supplementary material.

## References

[1] Stevens MM 2008 Biomaterials for bone tissue engineering. Mater. Today 11, 18–25. (10.1016/S1369-7021(08)70086-5)

[2] Wozney JM 2002 Overview of bone morphogenetic proteins. Spine 27, S2–S8. (10.1097/01.BRS.0000020725.01916.7E) 12205411

[3] Hoppe A, Guldal NS, Boccaccini AR 2011 A review of the biological response to ionic dissolution products from bioactive glasses and glass-ceramics. Biomaterials 32, 2757–2774. (10.1016/j.biomaterials.2011.01.004) 21292319

[4] Han P, Wu C, Xiao Y 2013 The effect of silicate ions on proliferation, osteogenic differentiation and cell signalling pathways (WNT and SHH) of bone marrow stromal cells. Biomater. Sci. 1, 379–392. (10.1039/C2BM00108J) 32481903

[5] Kim E-J, Bu S-Y, Sung M-K, Choi M-K 2013 Effects of silicon on osteoblast activity and bone mineralization of MC3T3-E1 cells. Biol. Trace Elem. Res. 152, 105–112. (10.1007/s12011-012-9593-4) 23306944

[6] Jones JR, Tsigkou O, Coates EE, Stevens MM, Polak JM, Hench LL 2007 Extracellular matrix formation and mineralization on a phosphate-free porous bioactive glass scaffold using primary human osteoblast (HOB) cells. Biomaterials 28, 1653–1663. (10.1016/j.biomaterials.2006.11.022) 17175022

[7] Xynos ID, Edgar AJ, Buttery LDK, Hench LL, Polak JM 2000 Ionic products of bioactive glass dissolution increase proliferation of human osteoblasts and induce insulin-like growth factor II mRNA expression and protein synthesis. Biochem. Biophys. Res. Commun. 276, 461–465. (10.1006/bbrc.2000.3503) 11027497

[8] Xynos ID, Edgar AJ, Buttery LDK, Hench LL, Polak JM 2001 Gene-expression profiling of human osteoblasts following treatment with the ionic products of Bioglass® 45S5 dissolution. J. Biomed. Mater. Res. 55, 151–157. (10.1002/1097-4636(200105)55:2<151::AID-JBM1001>3.0.CO;2-D) 11255166

[9] Gough JE, Jones JR, Hench LL 2004 Nodule formation and mineralization of human primary osteoblasts cultured on a porous bioactive glass scaffold. Biomaterials 25, 2039–2046. (10.1016/j.biomaterials.2003.07.001) 14741618

[10] Reffitt DM, Ogston N, Jugdaohsingh R, Cheung HFJ, Evans BAJ, Thompson RPH, Powell JJ, Hampson GN 2003 Orthosilicic acid stimulates collagen type 1 synthesis and osteoblastic differentiation in human osteoblast-like cells *in vitro*. Bone 32, 127–135. (10.1016/S8756-3282(02)00950-X) 12633784

[11] Maeno S, Niki Y, Matsumoto H, Morioka H, Yatabe T, Funayama A, Toyama Y, Taguchi T, Tanaka J 2005 The effect of calcium ion concentration on osteoblast viability, proliferation and differentiation in monolayer and 3D culture. Biomaterials 26, 4847–4855. (10.1016/j.biomaterials.2005.01.006) 15763264

[12] Honda Y, Fitzsimmons RJ, Baylink DJ, Mohan S 1995 Effects of extracellular calcium on insulin-like growth factor II in human bone cells. J. Bone. Miner. Res. 10, 1660–1665. (10.1002/jbmr.5650101108) 8592942

[13] Liu X, Ma PX 2004 Polymeric scaffolds for bone tissue engineering. Ann. Biomed. Eng. 32, 477–486. (10.1023/B:ABME.0000017544.36001.8e) 15095822

[14] Hutmacher DW, Schantz JT, Lam CXF, Tan KC, Lim TC 2007 State of the art and future directions of scaffold-based bone engineering from a biomaterials perspective. J. Tissue Eng. Regen. Med. 1, 245–260. (10.1002/term.24) 18038415

[15] Hutmacher DW 2000 Scaffolds in tissue engineering bone and cartilage. In The biomaterials: silver jubilee compendium (ed. Williams DF), pp. 175–189. Oxford, UK: Elsevier. 10.1016/s0142-9612(00)00121-6 11071603

[16] Obata A, Tokuda S, Kasuga T 2009 Enhanced *in vitro* cell activity on silicon-doped vaterite/poly(lactic acid) composites. Acta Biomater. 5, 57–62. (10.1016/j.actbio.2008.08.004) 18786869

[17] Obata A, Hotta T, Wakita T, Ota Y, Kasuga T 2010 Electrospun microfiber meshes of silicon-doped vaterite/poly(lactic acid) hybrid for guided bone regeneration. Acta Biomater. 6, 1248–1257. (10.1016/j.actbio.2009.11.013) 19913116

[18] Kasuga T, Obata A, Maeda H, Ota Y, Yao X, Kazuya O 2012 Siloxane-poly(lactic acid)-vaterite composites with 3D cotton-like structure. J. Mater. Sci.: Mater. Med. 23, 2349–2357. (10.1007/s10856-012-4607-5) 22415363

[19] Obata A, Ozasa H, Kasuga T 2013 Cotton wool-like poly(lactic acid)/vaterite composite scaffolds releasing soluble silica for bone tissue engineering. J. Mater. Sci.: Mater. Med. 24, 1649–1658. (10.1007/s10856-013-4930-5) 23606191

[20] Osada N, Makita M, Nishikawa Y, Kasuga T 2018 Cotton-wool-like resorbable bone void fillers containing β-TCP and calcium carbonate particles. Key Eng. Mater. 782, 53–58. (10.4028/www.scientific.net/KEM.782.53)

[21] Kenley RA, Lee MO, Mahoney TR, Sanders LM 1987 Poly(lactide-co-glycolide) decomposition kinetics *in vivo* and *in vitro*. Macromolecules 20, 2398–2403. (10.1021/ma00176a012)

[22] Mikos AG, Lyman MD, Freed LE, Langer R 1994 Wetting of poly(L-lactic acid) and poly(DL-lactic-co-glycolic acid) foams for tissue culture. Biomaterials 15, 55–58. (10.1016/0142-9612(94)90197-X) 8161659

[23] Loo SCJ, Tan ZYS, Chow YJ, Lin SLI 2010 Drug release from irradiated PLGA and PLLA multi-layered films. J. Pharm. Sci. 99, 3060–3071. (10.1002/jps.22079) 20112427

[24] Zhou P, Kasuga T 2018 Dissolution behavior of Mg/Si-doped vaterite particles in biodegradable polymer composites. Express Polymer Lett. 12, 171–179. (10.3144/expresspolymlett.2018.15)

[25] Zreiqat H, Howlett CR, Zannettino A, Evans P, Schulze-Tanzil G, Knabe C, Shakibaei M 2002 Mechanisms of magnesium-stimulated adhesion of osteoblastic cells to commonly used orthopaedic implants. J. Biomed. Mater. Res. 62, 175–184. (10.1002/jbm.10270) 12209937

[26] Wataha JC, Hanks CT, Craig RG 1992 *In vitro* synergistic, antagonistic, and duration of exposure effects of metal cations on eukaryotic cells. J. Biomed. Mater. Res. 26, 1297–1309. (10.1002/jbm.820261004) 1429749

[27] Zhai W, Lu H, Wu C, Chen L, Lin X, Kawazoe N, Chen G 2013 Stimulatory effects of the ionic products from Ca-Mg-Si bioceramics on both osteogenesis and angiogenesis *in vitro*. Acta Biomater. 9, 8004–8014. (10.1016/j.actbio.2013.04.024) 23619289

[28] Zhang X et al. 2015 Biodegradable mesoporous calcium–magnesium silicate-polybutylene succinate scaffolds for osseous tissue engineering. Int. J. Nanomed. 10, 6699–6708. (10.2147/IJN.S92598) PMC4630181 26604746

[29] Yamada S, Ota Y, Obata A, Kasuga T 2017 Osteoblast-like cell responses to ion products released from magnesium- and silicate-containing calcium carbonates. Bio-Med. Mater. Eng. 28, 47–56. (10.3233/BME-171655) 28269744

[30] Obata A, Ogasawara T, Yamada S, Kasuga T 2017 Combined effects of silicate, calcium and magnesium ions on osteoblast-like cell functions. Ceram. Eng. Sci. Proc. 37, 107–112. (10.1002/9781119321682)

[31] Obata A, Ogasawara T, Kasuga T 2019 Combinatorial effects of inorganic ions on adhesion and proliferation of osteoblast-like cells. J. Biomed. Mater. Res. A 107, 1042–1051. (10.1002/jbm.a.36623) 30684394

[32] Fujikura K, Lin S, Nakamura J, Obata A, Kasuga T 2013 Preparation of electrospun fiber mats using siloxane-containing vaterite and biodegradable polymer hybrids for bone regeneration. J. Biomed. Mater. Res. Part B 101, 1350–1358. (10.1002/jbm.b.32952) 23687079

[33] Nakamura J, Ota Y, Sakka Y, Kasuga T 2017 Interphase coordination design in carbamate-siloxane/vaterite composite microparticles towards tuning ion-releasing properties. Adv. Powd. Tech. 28, 1349–1355. (10.1016/j.apt.2017.03.003)

[34] Sepulveda P, Jones JR, Hench LL 2002 Characterization of melt-derived 45S5 and sol-gel-derived 58S bioactive glasses. J. Biomed. Mater. Res. 58, 734–740. (10.1002/jbm.10026) 11745528

[35] Hench LL 2013 Chronology of bioactive glass development and clinical applications. New J. Glass Ceram. 3, 67–73. (10.4236/njgc.2013.32011)

[36] Kargozar S, Baino F, Hamzehlou S, Hill RG, Mozafari M 2018 Bioactive glasses entering the mainstream. Drug Discov. Today. 23, 1700–1704. (10.1016/j.drudis.2018.05.027) 29803626

[37] Kasuga T, Maeda H, Kato K, Nogami M, Hata K, Ueda M 2003 Preparation of poly(lactic acid) composites containing calcium carbonate (vaterite). Biomaterials 24, 3247–3253. (10.1016/S0142-9612(03)00190-X) 12763452

[38] Lin CC, Chen SF, Leung KS, Shen P 2012 Effects of CaO/P_2_O_5_ ratio on the structure and elastic properties of SiO_2-_CaO-Na_2_O-P_2_O_5_ bioglasses. J Mater. Sci: Mater. Med. 23, 24–258. (10.1007/s10856-011-4504-3) 22109904

[39] Silva AMB, Queiroz CM, Agathopoulos S, Correia RN, Fernandes MHV, Oliveira JM 2011 Structure of SiO_2_-MgO-Na_2_O glasses by FTIR, Raman and ^29^Si MAS NMR. J. Mol. Struct. 986, 16–21. (10.1016/j.molstruc.2010.11.023)

[40] Trcera N, Rossano S, Tarrida M 2011 Structural study of Mg-bearing sodosilicate glasses by Raman spectroscopy. J. Raman Spectrosc. 42, 765–772. (10.1002/jrs.2763)

[41] Watts SJ, Hill RG, O'Donnell MD, Law RV 2010 Influence of magnesia on the structure and properties of bioactive glasses. J. Non-Cryst. Solids 356, 517–524. (10.1016/j.jnoncrysol.2009.04.074)

[42] Jones JR 2013 Review of bioactive glass: from Hench to hybrids. Acta Biomater. 9, 4457–4486. (10.1016/j.actbio.2012.08.023) 22922331

